# Ultra-Processed Food Consumption Among College Students and Their Association With Body Composition, Bowel Movements and Menstrual Cycle

**DOI:** 10.3389/ijph.2025.1607712

**Published:** 2025-04-08

**Authors:** Anindita Ghosh, Arti Muley

**Affiliations:** ^1^ Department of Nutritional Sciences and Dietetics, Symbiosis Skills and Professional University, Pune, Maharashtra, India; ^2^ Department of Nutrition and Dietetics, Symbiosis School of Culinary Arts and Nutritional Sciences (SSCANS), Symbiosis International (Deemed University), Pune, Maharashtra, India

**Keywords:** ultra-processed food choices, body composition, body mass index, bowel movements, irregular menstrual cycle

## Abstract

**Objectives:**

The current research aimed to explore the association of ultra-processed food consumption among college students with body composition, bowel movements, and menstrual irregularities with a focus on females.

**Methods:**

A cross-sectional study was conducted in Pune, India among 110 university students of both genders aged 18–25 years. A developed and validated Food Frequency Questionnaire (FFQ) based on the NOVA classification was used to evaluate UPF consumption, while the Constipation Scoring System (CSS) and the Premenstrual Symptoms Screening Tool assessed bowel habits and menstrual health, respectively. Anthropometric measurements, including BMI, body fat percentage,and visceral fat were recorded using an Omron Karada Analyzer.

**Results:**

A higher percentage of participants were female (74.8%). Most participants (52.3%) consumed more than three meals daily, while 42.1% ate outside food 2–3 times per week. Higher UPF consumption showed a trend toward increased body fat (p = 0.053) and was significantly associated with greater visceral fat accumulation (p < 0.05). No significant associations were found between UPF intake and bowel movement, gastrointestinal symptoms, or menstrual cycle irregularities (p > 0.05).

**Conclusion:**

Higher UPF consumption showed a trend toward increased body fat percentage, though not statistically significant. These findings highlight the need to reduce UPF intake to mitigate potential risks of increased adiposity and metabolic disturbances.

## Introduction

The reports on food adulteration have increased, and the consumption of highly processed and ready-to-eat items has grown. The consumption of such food has contributed to rising obesity rates globally and in India [1]. Teenagers and young adults are often the most affected by changing food habits, social pressures, and socioeconomic factors [2].

A balanced diet is essential for energy and provides macronutrients, essential fats, amino acids, vitamins, and minerals [3]. Improper nutrient intake can harm health, leading to issues like hair loss, brittle nails, and weight loss [4]. Excessive intake of fast foods high in fats, sugars, and sodium contributes to weight gain and various health issues, including obesity [5].

An ultra-processed food (UPF) is created primarily for commercial gain, designed to be hyper-palatable. These foods often include organic or synthetic additives, preservatives, and colorings to enhance taste and appeal. Industrial processing methods like frying, freezing, and hydrogenation are typically used in their production. Avoiding UPF among teenagers and young adults is crucial for protecting future generations. Poor nutrition can lead to chronic health issues like Type 2 diabetes, obesity, and cardiovascular disease [6].

Understanding UPF consumption among college students is necessary for their health and wellbeing [7]. Poor dietary consumption among college students can impact their health and academic performance [8]. Reports indicate that university students experience a significant shift in lifestyle, greatly affecting their dietary habits [9]. Most college students usually eat in a mess and college canteen facilities availing limited or routine healthy eating options [10]. Other major factors influencing their dietary consumption are availability of time and financial conditions [11]. Limited time and a fixed budget make it hard to balance studies, extracurriculars, cooking, and cleaning, leading many to rely on mess services, street food, or convenience items for affordability and convenience [12]. Young adults and teenagers face higher disease risk due to increased consumption of processed foods high in fat, sugar, sodium, and preservatives, along with reduced intake of fresh, nutritious meals [13]. Moreover, the availability of UPF is higher and cheaper as compared to healthy homemade meals [14]. Enhanced taste also induces UPF consumption among students [15]. A repetitive menu with limited taste in the mess food also provokes students to have ultra-processed foods [7]. UPF consumption among students are influenced by social, psychological, and financial factors such as mood, cravings, quality, pricing, preferences, and eating behaviors [16].

India’s diverse religious and cultural landscape influences ultra-processed food (UPF) consumption and dietary behaviours. Thus, a wide range of consumption patterns across the population is observed [15]. Students relocating for higher studies often have difficulty adjusting to new food tastes and ingredients, resulting in a reliance on ultra-processed foods to satisfy cravings [17]. Studies indicate that male students often skip breakfast and rely on outside foods, while female students are more likely to prepare meals and include some fruits and vegetables in their diets [18].

Irregular meal timings, high intake of fats and sugars, and lack of physical activity among students contribute significantly to global obesity and related health issues, including impaired bowel movements [19]. Early onset of chronic diseases related to obesity and overweight in adults also negatively affects students’ academic performance [20]. Rising academic stress, lifestyle changes, and cheap fast food availability have led to unhealthy diets, increasing obesity, and nutritional disorders in teenagers and adults [21].

Diet influences the body composition of individuals, including weight-to-height ratios and the percentage of body fat index (PBF) [5]. Higher consumption of calorie-dense foods like French fries and candies is linked to increased PBF and BMI, while students who eat more fruits and vegetables have lower values for these metrics [22]. Many students snack on high-fat and sugary foods, a significant cause of weight gain, regardless of their health conditions [23]. Healthy eating habits reflect healthy and normal body composition indicators like adipose tissue, BMI, and body type [7].

An unhealthy diet may cause common health problems such as gastric issues including acidity, bloating, and constipation [24]. Students are experiencing constipation regularly due to low consumption of fruits, and vegetables, and high intake of sugar, fat, and refined products [25]. UPF consumption are associated with constipation as they have a high impact on the colon [26]. Also, unhygienic food habits and high consumption of sugary foods have been associated with diarrhoea [27]. Low-fibre diets can disrupt digestion, causing constipation, diarrhoea, and other gastric issues, and may increase the risk of inflammatory and irritable bowel disorders over time [28]. Irregular bowel movements can impact gut health and mood, leading to symptoms like headaches, coated tongue, foul breath, and depression. This can significantly reduce the productivity and wellbeing of students and individuals [2].

Rising cases of polycystic ovarian disease (PCOD) and female infertility were found associated with UPF consumption from childhood to infancy [20]. Pre-menstrual syndrome (PMS) symptoms are linked to unhealthy food consumption. In female students, high caffeine intake, fried foods, sweets, zero-calorie drinks, smoking, lack of physical activity, and higher BMI are associated with increased insulin resistance and worsened PMS symptoms [13, 29]. Obesity is a major risk factor for various diseases like type 2 diabetes mellitus and dyslipidaemia, and it has adverse effects on iron status [11]. Mindful eating and a balanced diet promote long-term health. Educating students about avoidance of UPF and implementing proper nutrition practices are essential [24].

The study evaluated how UPF consumption among college students affects body composition, bowel movement, and PMS symptoms.

## Methods

### Study Design and Participants

**FIGURE 1:**
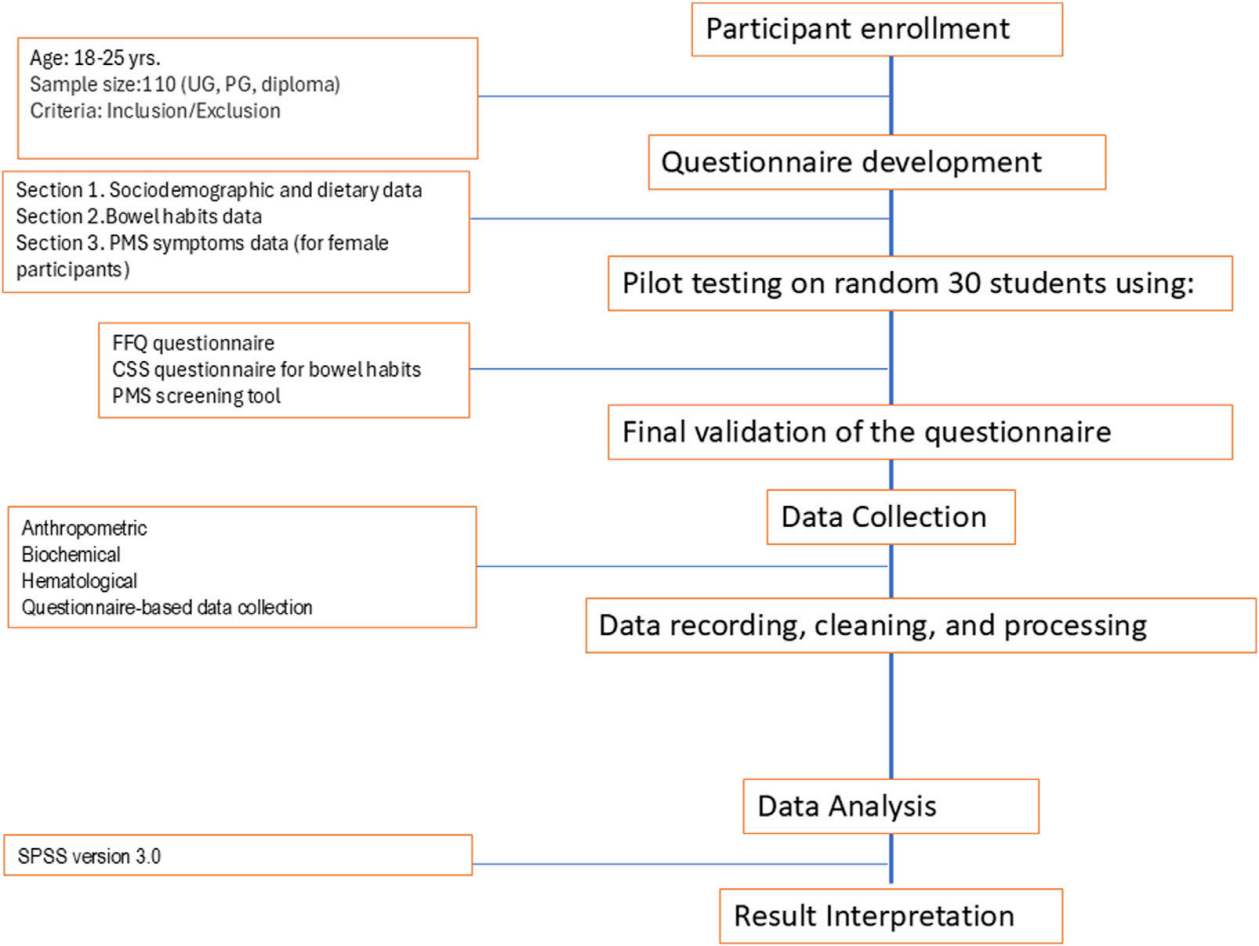
Flow chart of the study (Ultra-Processed Food Consumption Among College Students and Their Association With Body Composition, Bowel Movements and Menstrual Cycle, Pune, India. 2024).

This cross-sectional study targeted students aged between 18 and 25 years enrolled at a University in Pune, India. A non-random sampling technique was employed to recruit 110 diploma, undergraduate, and postgraduate students both genders over 3 months. The study followed a two-phase approach with inclusion criteria specifying students aged 18–25 years without known health issues (Figure 1). Exclusion criteria included students with health conditions, bleeding disorders, refusal to consent, or those enrolled at other institutions. Ethical approval was obtained from Symbiosis Skills and Professional University (SSPU/R/2024/461) following the institutional guidelines. Written informed consent was obtained from all participants before their involvement in the study. This study was conducted between April 2024 and July 2024.

Each study participant had a face-to-face interview to answer the questionnaire, where each student took 20–30 min to complete the questionnaire and to perform the physical measurements privately in the presence of the researchers. A trained nurse performed the haemoglobin assessments as a part of the participant health screening, rather than as a study variable. BMI was classified based on the World Health Organization (WHO) guidelines: participants with a BMI below 25 kg/m^2^ were categorized as normal body weight, those with a BMI ranging from 25 to 29 kg/m^2^ were classified as overweight, and a BMI of 30 kg/m^2^ or higher was defined as obesity.

### Phase 1: Questionnaire Development

A structured questionnaire was developed and validated to collect data, covering socio-demographic details, dietary habits, UPF consumption, gastrointestinal health, and premenstrual syndrome (PMS) symptoms. The questionnaire consisted of three sections:

1. Sociodemographic and dietary assessment: Included questions on age, gender, weight, height, frequency of engagement in physical activity, and dietary consumption, assessed using a validated Food Frequency Questionnaire (FFQ) to measure the frequency of UPF consumption.Reference for UPF Classification: The validated Food Frequency Questionnaire (FFQ) with frequency categories ranging from daily to never was used for assessing UPF consumption. This questionnaire was developed and then validated based on the NOVA classification system [30], which categorizes food according to its level of processing. This classification method ensured a standardized evaluation of ultra-processed food intake.
2. Bowel movement assessment: Utilized the Constipation Scoring System (CSS) to evaluate bowel habits and stool frequency
3. Menstrual health assessment: The Premenstrual Symptoms Screening Tool applicable to female participants, was used to document the menstrual cycle irregularities and symptoms related to PMS.

The questionnaire was adapted from previously validated tools ensuring reliability in assessing dietary consumption and health indicators. A pilot testing was done with 30 participants to refine clarity and relevance, feedback was incorporated into the final version before full-scale data collection started.

### Phase 2: Data Collection

Anthropometric data, dietary intake, physical activity, bowel habits, and PMS symptoms were collected. Height was measured with a stadiometer; weight, BMI, body fat, and visceral fat were estimated using an Omron Karada analyser.

### Data Analysis

Statistical analysis were performed using SPSS Version 3.0. Descriptive statistics, including means and standard deviations, were calculated for anthropometric and dietary variables. Pearson’s correlation test was used to examine the relationship between UPF consumption and body composition, bowel movements, and menstrual cycle. A p-value of <0.05 was considered statistically significant.

## Result

### Demographic Characteristics and Dietary Assessment

A comprehensive survey of 110 students was done and we assessed dietary habits, encompassing age, gender, living arrangements, meal patterns, water intake, dietary behaviours, and physical activity levels. Although the study was conducted in both genders; most participants were female (74.8%) and belonged to the 18–25 years age group. A majority (72%) were day scholars, while 28% lived in hostels. In meal preparation, 61.7% cooked their meals, while 38.3% used external food sources like mess services.

### Meal Frequency and Dietary Patterns

We analyzed meal frequency, outside food consumption, water intake, and breakfast eating habits of the participants. Results indicated that 52.3% consumed more than three meals daily, while 25.2% had two or three meals. Regarding outside meals, 42.1% ate out 2–3 times weekly, and 38.3% did so once a week. Water intake showed that 44.9% consumed 2–3 L daily, with 20.6% exceeding 3 L, indicating good hydration. For breakfast, 35.5% never skipped it, while 19.6% always did.

### Physical Activity

Physical activity levels varied among the participants, with 35.5% being inactive, 35.5% engaging in daily exercise, and 39.3% exercising 2–3 times weekly. These findings indicate a significant proportion of the participant students incorporated regular physical activity into their routines, although a significant percentage remained inactive.

### Dietary Consumption Patterns

The dietary habits of respondents were further assessed in terms of food consumption frequency (Supplementary File S1). A significant proportion reported consuming fresh fruits (54.7%) and milk/milk products (50.5%) daily. The intake of cooked and raw vegetables was lower, with 37.9% consuming them daily. Interestingly, 34.7% of participants never consumed meat, while 36.8% never consumed eggs, highlighting notable dietary exclusions. Alcohol consumption was relatively low, with 77.9% of respondents abstaining completely.

### Ultra Processed Food Consumption Patterns

To analyze ultra-processed food (UPF) consumption, participants rated various UPF items on a scale of 1–5, where 1 indicated the lowest consumption and 5 the highest (Table 1). The results showed that chocolates (23.2%) and ice cream (25.5%) were among the most frequently consumed UPFs.Conversely, mayo (38.3%) and soft drinks (33.7%) had lower reported consumption. These findings suggest variability in UPF intake, with some products being more commonly consumed than others.

**TABLE 1:** Consumption patterns of ultra-processed food items among the participants (Ultra-Processed Food Consumption Among College Students and Their Association With Body Composition, Bowel Movements and Menstrual Cycle, Pune, India. 2024).

Ultra processed food item	Lowest rating % [1]	Highest rating [5]
Burger	26.3	17.9
Pizza	22.1	22.1
Samosa	24.2	6.3
Vada Pav	18.9	13.7
Noodles	28.4	12.6
Packet/Ready to Eat Foods	30.5	9.5
Biscuits	24.2	5.3
Cakes/Pastries	16.1	17.2
Soft Drinks	33.7	8.4
Bread and Bakery Items	14.7	9.5
Mayo	38.3	7.4
Ketchup/Sugar-Based Sauces/Dips	31.9	7.4
Chocolates	14.7	23.2
Ice Cream	14.9	25.5
Chat	17.0	24.5

Table 1 illustrates participants’ ratings, revealing their tendencies toward different ultra-processed foods and offering insights into consumption trends and dietary inclinations.

### Relationship Between UPF Consumption and Body Composition

The anthropometric analysis of participants revealed a mean height of 159 cm, weight of 58 kg, and BMI of 22.4, placing most individuals within normal and overweight categories. The mean percentage of body fat index (PBF) was 28%, indicating a tendency towards higher adiposity. Table 2 presents the relationship between UPF consumption and body composition, analyzed using ANOVA. Food intake was categorized based on the NOVA classification system, which groups foods by processing level. Unprocessed or minimally processed foods, such as fresh fruits, vegetables, whole grains, plain dairy, and unseasoned lean meats were identified as healthy food categories. Ultra-processed foods (UPFs) were identified as foods, such as packaged snacks, sugary-sweetened beverages, instant noodles and processed meats that are industrially formulated food products containing additives [30]. The result showed that while healthy food consumption did not significantly affect body composition (p = 0.479), UPF consumption exhibited a trend towards significance (p = 0.053), suggesting a potential association between frequent UPF intake and increased body fat.

**TABLE 2:** The relationship between ultra-processed food consumption and body composition (Ultra-Processed Food Consumption Among College Students and Their Association With Body Composition, Bowel Movements and Menstrual Cycle, Pune, India. 2024).

Food Consumption Type	Between and Within Groups	Sum of Squares	df (degrees of freedom)	Mean Square	F-value	Sig.(p-value)
Healthy Food	Between Groups	53.427	2	26.714	0.75	0.479
	Within Groups	1,281.496	36	35.597		
	Total	1,334.923	38			
UPF	Between Groups	1,178.355	2	589.177	3.196	0.053
	Within Groups	6,636.722	36	184.353		
	Total	7,815.077	38			

Although the observed association did not reach the full statistical significance, the F-value of 3.196 for UPF consumption indicates notable variability in body composition among different intake groups. These findings suggest that higher UPF consumption may contribute to increased fat accumulation, highlighting the need for further study to explore its long-term metabolic effects. Given the growing reliance on UPFs, understanding their role in adiposity and body composition changes over time remains critical for promoting healthy dietary habits.

### Relationship Between UPF Consumption and Visceral Fat

Dietary patterns, particularly the consumption of UPFs play a crucial role in body fat distribution and metabolic health. In this study, a significant positive correlation was observed between UPF consumption and visceral fat levels (p < 0.05), indicating that individuals with high UPF intake tended to have greater visceral fat accumulation.

### Relationship Between UPF Consumption and Gastrointestinal Symptoms and Bowel Movement

Ultra-processed food (UPF) consumption has been suggested to influence digestive health, particularly bowel movement patterns. This study examined the association between UPF intake and common digestive issues such as constipation, bloating, and incomplete evacuation. However, the findings indicated no significant correlation between UPF consumption and bowel movement irregularities (p > 0.05) as shown in Table 3.

**TABLE 3:** Correlation between ultra-processed food consumption and gastrointestinal symptoms and bowel movement (Ultra-Processed Food Consumption Among College Students and Their Association With Body Composition, Bowel Movements and Menstrual Cycle, Pune, India. 2024).

Gastrointestinal symptoms and bowel movement	Food consumption type	p-value
How often do you suffer from constipation	Healthy food	0.846
	UPF^a^	0.843
How often do you suffer from bloating and acidity	Healthy food	0.860
	UPF	0.307
How often do you experience incomplete evacuation	Healthy food	0.676
	UPF	0.475

^a^ UPF: Ultra-Processed Foods, classified according to [30]

No correlations were found to be statistically significant at *p* < 0.05.

Constipation frequency did not differ significantly between individuals consuming healthy foods and those consuming UPFs (p = 0.843). Similarly, bloating and acidity were not significantly associated with UPF intake (p = 0.307), nor was the sensation of incomplete evacuation (p = 0.475). These results suggest that UPF may not have an immediate or direct association with bowel movement patterns among the study participants.

### Relationship Between UPF Consumption and Menstrual Cycle

The association between UPF consumption and menstrual patterns was analysed among female respondents to assess whether dietary habits influenced menstrual regularity and premenstrual symptoms (PMS). The findings indicated no significant relationship (p > 0.05) between UPF consumption and menstrual cycle regularity, suggesting that frequent intake of UPF does not directly associate with the timing or consistency of the menstrual cycles. Similarly, common menstrual symptoms such as back pain and fatigue were not significantly linked to dietary patterns, indicating the food choices-whether healthy or ultra-processed -did not influence these discomforts. Although many participants reported experiencing food cravings before menstruation, these cravings did not show a statistically significant correlation with either healthy food consumption (p = 0.838) or UPF consumption (p = 0.232). Table 4 summarizes these findings, reinforcing that UPF intake had no notable association with menstrual cycle regularity, symptoms, or cravings.

**TABLE 4:** Correlation between ultra-processed food Consumption and Menstrual Cycle (MC) Regularity and premenstrual symptoms (Ultra-Processed Food Consumption Among College Students and Their Association With Body Composition, Bowel Movements and Menstrual Cycle, Pune, India. 2024).

Menstrual cycle (MC) regularity & PMS symptoms	Food consumption type	p-value
MC regularity	Healthy food	0.443
	UPF^a^	0.698
Which symptoms do you experience in menstruation	Healthy food	0.617
	UPF	0.969
Do you experience food cravings before menstrual cycle	Healthy food	0.838
	UPF	0.232

^a^ UPF: Ultra-Processed Foods, classified according to [30]

No correlations were found to be statistically significant at p < 0.05.

## Discussion

The findings suggest that UPF consumption, while not significantly affecting BMI—most respondents were in the normal to overweight range—strongly influence PBF, with an average of 28%, exceeding healthy levels. This highlights BMI’s limitations for not differentiating fat from lean mass, making it an inadequate measure for detailed nutritional or gender-specific body composition assessments [29]. PBF and visceral fat levels are key markers for linking UPF consumption to body composition. With a mean PBF of 28% ± 5.01%, many female participants approached or exceeded the recommended 15%–30% range, indicating excess fat likely influenced by UPF consumption, enhancing future obesity risk [21]. Visceral fat, a key health marker (4% ± 4%) was generally within healthy limits. However, dietary patterns revealed insufficient protein intake, with 36% never consuming eggs and 34% avoiding chicken, indicating potential protein deficiencies, especially among vegetarians. Only 37.9% reported daily fruit consumption, far below recommended levels, risking micronutrient deficiencies.

These findings highlight significant nutritional gaps with potential long-term health risks [7]. The FFQ questionnaire highlighted a preference for ultra-processed foods such as chocolates (25%), ice creams, and chaat (24% each), followed by pizzas (22%), burgers (17%), and noodles (12%). These preferences reflected a reliance on UPF over freshly prepared meals, driven by time constraints and stressful lifestyles. This dietary pattern correlates with elevated body fat percentages (PBF) due to the high fat and low food nutrient content. Additionally, 42% of participants ordered food 2–3 times weekly, while 35% reported no physical activity, contributing to visceral fat accumulation and metabolic health risks.

These findings suggest students rely heavily on outside food and exhibit low physical activity levels, resulting in higher PBF and visceral fat percentages. Such habits increase susceptibility to metabolic disorders, underscoring the need for targeted dietary and lifestyle interventions [31].

### UPF Consumption and Its Association With Bowel Movements

**FIGURE 2:**
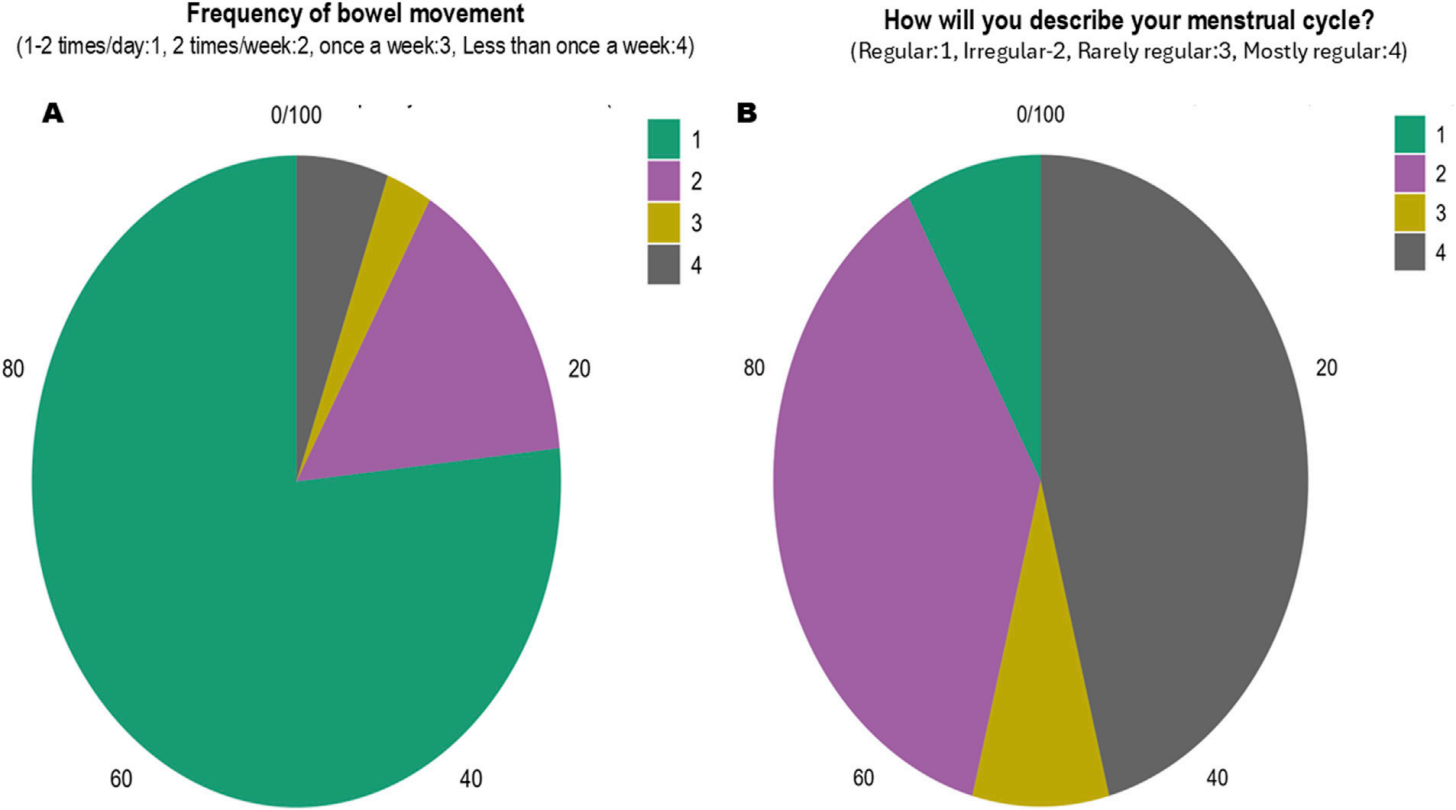
Presentation of the questionnaire-based survey results on **(A)** the frequency of bowel movement, and **(B)** reported menstrual cycle conditions by the female participants (Ultra-Processed Food Consumption Among College Students and Their Association With Body Composition, Bowel Movements and Menstrual Cycle, Pune, India. 2024).

The dietary patterns and their relation with bowel movements were studied (Figure 2A). Sugar items and high-lipid foods have been linked with constipation as compared to grains, fruits, and vegetables [6]. Most participants (76.6%) reported bowel movements (BM) 1–2 times daily, while 15% had BM twice weekly, 2.8% had BM once weekly, and 5.6% less than once weekly.

Those with infrequent BM were often linked to never consuming grains like cereals and pulses (8%). Although 37% of respondents consumed raw or cooked vegetables, fibre intake remained low overall.

Regarding constipation, 35.5% reported never experiencing it, 41.1% experienced it rarely, 18.7% experienced it sometimes, and 4.7% experienced it always. Constipation was more prevalent among those favouring low-fibre, high-sodium ultra-processed foods such as pizza, burgers, and noodles, which are associated with incomplete evacuation and unsatisfactory bowel movements. These findings highlight the role of dietary habits in gastrointestinal health and the potential risks of UPF consumption [7]. Low intake of soluble fibre has been associated with feelings of incomplete evacuation. Among respondents, 37.4% reported never experiencing incomplete evacuation, 39.3% mentioned rare incomplete evacuation, 19.6% mentioned it sometimes, and 3.7% mentioned it to experience always. These symptoms may correlate with high consumption of sugary items like cakes and pastries (17% preference) and ready-to-eat foods such as noodles (22% preference).

Adequate daily intake of soluble and insoluble fibres is crucial for reducing gastrointestinal issues while incorporating prebiotics and probiotics is known to support gut health and improve digestive function [26]. Around 24.3% of respondents reported never experiencing acidity or bloating, while 40.2% experienced it rarely, 30.8% experienced it sometimes, and 4.7% always experienced it. These issues were more common among participants with low soluble fibre intake and high consumption of sugary items like cakes and pastries. Longer meal gaps and sedentary lifestyles also exacerbated these problems. Notably, 25% of respondents consuming two or fewer meals per day and 19% skipping breakfast had higher occurrences of bloating and acidity.

Regarding supplements, 75.7% never used bran fibre or laxatives for constipation relief. Analysis revealed no significant differences in bowel movement frequency (F-ratio = 0.750, p = 0.479) or acidity/bloating issues related to ultra-processed food consumption (F-ratio = 0.252, p = 0.860). Despite this, further research focusing on daily food intake, meal size, and meal portion proportions is recommended to understand these relationships.

### UPF Consumption and Its Association With PMS Symptoms

Our study found that 37.6% of respondents had regular menstrual cycles, while 45.9% reported irregular cycles (Figure 2B). The population with irregular cycles correlated with high consumption of fat- and sugar-rich foods, suggesting that UPF consumption may impact menstrual health and PMS symptoms. We noted that 27% of the participants regularly skipped breakfast, and 48% skipped it frequently.

Previous research links inconsistent breakfast consumption with a higher occurrence of menstrual disorders in teenage girls [32]. This highlights the potential influence of eating habits on menstrual cycle regularity [33]. Along with UPF consumption, irregularity in taking meals can also lead to a disrupted menstrual pattern. A family history of menstrual abnormalities can lead to irregular menstrual cycles due to genetic defects, 91.9% reported no family history of menstrual abnormalities, while only 8.1% reported having such a family history. This refers to the fact that most of the irregular menstrual cycles in the given study are associated with increased UPF consumption among the female population. Abbas et al. further concluded that dysmenorrhea, fatigue, irritability, and anxiety were the most common symptoms of PMS experienced by women [34].

In this study, the most commonly reported symptoms before the MC were cramps (16.9%) and mood swings (20.5%). Back pain (13.3%) and fatigue (2.4%) were also reported, and a significant percentage (47.0%) reported experiencing all of the above symptoms. These commonly occurring symptoms before the MC are usually seen in females lacking physical activity. We noted that 27% of the participants did not engage in any physical activity, and 42% engaged in physical activity twice or thrice a week. These elevated symptoms during PMS can be linked to lower physical activities among respondents. Connecting with lifestyle, 75.0% reported experiencing food cravings during or before their menstrual cycle, while 25.0% reported no food cravings. Among the respondents, 53.7% reported feeling changes in their premenstrual syndrome (PMS) due to the consumption of high-fat, high-sugar foods, which included severe to moderated acne breakdown, while 46.3% reported no such changes. Similarly, Bancroft et al. observed that 72% of the female participants reported premenstrual food cravings [32].

The study findings suggest no significant association between UPF consumption and menstrual irregularities or premenstrual symptoms (PMS). Analysis of menstrual abnormalities based on healthy or UPF consumption yielded F-ratios of 0.157 and 0.079 (p > 0.05), indicating no significant role of these dietary consumption in menstrual irregularities. Similarly, symptoms such as cramps, mood swings, fatigue, and back pain reported before menstruation showed no significant variation based on food consumption (p > 0.05). Food cravings during or before the MC were unaffected by dietary consumption, as reflected in F-ratios of 0.046 and 1.417. These findings suggest that UPF consumption has minimal association with menstrual health and related symptoms. However, potential biases and confounding factors, such as meal proportions, symptom severity, genetic predispositions, or medical conditions, warrant further investigation through more detailed studies.

### Conclusion

UPF consumption may not significantly be associated with BMI but are associated with higher PBF. This highlights BMI’s limitations as a standalone indicator of body composition, particularly for women with higher body fat levels. While the average visceral fat level was at a healthy level, many individuals relying heavily on ultra-processed meals and engaging in little physical activity showed potential for excessive fat accumulation. Among vegetarians, inadequate intake of fruits, vegetables, and protein was found associated with increased body fat and nutritional deficiencies.

Ultra-processed foods were also associated with digestive issues, such as bloating and constipation, although changes in bowel frequency were not statistically significant. Irregular menstruation was associated with high fat and sugar intake and skipping meals.

These findings underscore the multifaceted impacts of ultra-processed foods and the need to integrate body composition and physiological markers alongside BMI in nutritional assessments.

## Data Availability

Data will be available from the authors on request.

## Abbreviations

UPF, Ultra-Processed Food; PBF, Percentage of Body Fat; PMS, Pre-Menstrual Syndrome; MC, Menstrual Cycle; BM, Bowel Movement.
