# Supplementary material for: Ultra-Processed Food Consumption Among College Students and Their Association With Body Composition, Bowel Movements and Menstrual Cycle
Source: Int J Public Health. 2025 Apr 8;70:1607712. doi: 10.3389/ijph.2025.1607712 (PMC12011617; doi:10.3389/ijph.2025.1607712)
Supplement: Supplementary file 1 [file Table1.docx]

| **Food Groups and Dietary Components** | **Consumption Frequency (Minimal and Null)** | **Percentage of Respondents** |
| --- | --- | --- |
| Cereals and Pulses | Once a day or more | 38.9% |
|  | Never | 8.4% |
| Fresh Fruits | Once a day or more | 54.7% |
|  | Never | 4.2% |
| Cooked/Raw Vegetables | Once a day or more | 37.9% |
|  | Never | 5.3% |
| Meat and Chicken | 1-2 times a week | 41.1% |
|  | Never | 34.7% |
| Egg | 1-2 times a week | 34.7% |
|  | Never | 36.8% |
| Milk and Milk Products | Once a day or more | 50.5% |
|  | Never | 4.2% |
| Tea/Coffee | Once a day or more | 49.5% |
|  | Never | 22.1% |
| Alcohol | Never | 77.9% |
|  | Once a fortnight | 12.6% |

**Supplementary Table 1. Consumption frequency of various food groups among the respondents** **(Pune, India. 2024).**

Table 1 provides a summary of respondents' minimal and null consumption frequencies for various food items, highlighting common dietary patterns.
